# Overview of international naturopathic practice and patient characteristics: results from a cross-sectional study in 14 countries

**DOI:** 10.1186/s12906-020-2851-7

**Published:** 2020-02-18

**Authors:** Amie Steel, Hope Foley, Ryan Bradley, Claudine Van De Venter, Iva Lloyd, Janet Schloss, Jon Wardle, Rebecca Reid

**Affiliations:** 1 Australian Research Centre in Complementary and Integrative Medicine, Faculty of Health, University of Technology Sydney, Level 8, Building 10, 235-253 Jones St, Ultimo, NSW 2006 Australia; 2 Office of Research, Endeavour College of Natural Health, Brisbane, Australia; 3 Helfgott Research Institute, National University of Natural Medicine, Portland, OR USA; 4 Division of Preventive Medicine, University of California, San Diego, La Jolla, CA USA; 5 World Naturopathic Federation, Toronto, ON Canada

**Keywords:** Naturopathy, Primary care, Health services research, Survey, Practice behaviours

## Abstract

**Background:**

Naturopathy is a distinct system of traditional and complementary medicine recognized by the World Health Organization and defined by its philosophic approach to patient care, rather than the treatments used by practitioners. Worldwide, over 98 countries have practicing naturopaths, representing 36% of all countries and every world region. The contributions of naturopaths to healthcare delivery services internationally has not been previously examined. Thus, the primary intention of this research was to conduct an international survey of naturopathic practice and patient characteristics in order to gain insight to the breadth of their practices and the type of clinical conditions routinely encountered.

**Methods:**

The cross-sectional study was conducted in naturopathic clinics in 14 countries within 4 world regions including the European (Portugal, United Kingdom, Switzerland, Spain), Americas (Canada, United States, Chile, Brazil), Western Pacific (Hong Kong, Australia, New Zealand) and African (South Africa). Naturopathic practitioners in each country were invited to prospectively complete an online survey for 20 consecutive cases. The survey was administered in four languages.

**Results:**

A total of 56 naturopaths from 14 countries participated in the study, providing a mean of 15.1 cases each (SD 7.6) and 851 cases in total. Most patients were female (72.6%) and all age categories were represented with a similar proportion for 36–45 years (20.2%), 46–55 years (19.5%), and 56–65 years (19.3%). A substantial majority (75%) of patients were considered by the participant to be presenting with chronic health conditions. The most prevalent category of health conditions were musculoskeletal (18.5%), gastrointestinal (12.2%), and mental illness (11.0%). The most common treatment categories prescribed or recommended to patients by the participants were dietary changes (60.5%), lifestyle and behaviour changes (56.9%), herbal medicines (54.2%) and nutritional supplements (52.1%). Many patients were known by participants to be receiving care from a general practitioner (43.2%) or a specialist medical practitioner (27.8%).

**Conclusions:**

Naturopathic practitioners provide health care for diverse health conditions in patients in different age groups. The global population would benefit from researchers and policy makers paying closer attention to the potential risks, benefits, challenges and opportunities of the provision of naturopathic care within the community.

## Introduction

Naturopathy is a distinct system of traditional and complementary medicine (T&CM) recognized by the World Health Organization (WHO) [1]. The World Naturopathic Federation (WNF), established as an internationally representative body for the naturopathic profession globally [2], defines naturopathy as a system of healthcare with a deep history of traditional philosophies and practices, medically trained practitioners and a breadth of natural treatment options to serve patients [3]. In many countries, the educational model for naturopathy is comparable to biomedical training with its foundation in anatomy, physiology and diagnostics. Naturopathic clinical education emphasizes non-drug based treatments including lifestyle-oriented self-care; preventive behaviors, dietary nutrition, physical activity, and stress-management counseling; clinical nutrition (i.e., targeting pharmacologic actions by nutrients for specific diseases irrespective of nutrient status); herbal medicine; homeopathy and hands-on manual therapies, more so than over-the-counter and prescription drug therapies or surgical interventions [4–12].

Notably, although naturopaths have unique training in treatments not represented in other areas of medicine, the profession is defined more by its philosophic approach to patient care, rather than the treatments used. The philosophy has been codified into seven principles including: *First Do No Harm*; *Doctor as Teacher*; *Apply the Healing Power of Nature*; *Treat the Whole Person*; *Treat the Cause*; *Wellness*; *Health Promotion and Disease Prevention* [3]. These principles provide a conceptual model for patient encounters, including a comprehensive consultation and examination process, common approach to clinical diagnostic processes, and the application of various treatments. Worldwide, 98 countries are known to have naturopathic practitioners, representing every world region [13]. The largest proportion of countries in any world region where naturopathic practitioners are providing care is North America (67%) [14], and this region also has one of the more established regulatory positions for naturopaths [15]. In North America, where much of the early professional formation of the naturopathic profession was centered, naturopathy is licensed in six Canadian provinces and 20 US states as well as the Washington District of Columbia (D.C.) and the territories of Puerto Rico and the U.S. Virgin Islands [16, 17]. In Europe, where naturopathy and its precursors (such as nature cure) originate and have been practiced for centuries, naturopathic practitioners are reported in over 30 European countries [15], only three of which regulate naturopathic practice [15]. Only a slightly lower proportion of countries in Latin America (43%) have naturopathic practitioners [14]. In Asia, naturopathic practitioners have a significant presence in India, Nepal, Hong Kong, Indonesia, Malaysia, Singapore and Thailand while naturopathic practitioners are also one of the dominant traditional medicine systems in Australia and New Zealand [14].

Although several evaluations of naturopathic practice (including prospective clinical trials and retrospective practice audits) suggest favorable contributions of naturopaths to both patient health outcomes, and established measures of primary care quality [6–8, 11, 18, 19] the contributions of naturopaths to healthcare delivery services internationally has not been previously examined via formal research. Given many health conditions remain challenging to manage, either due to limitations in available treatments (e.g., chronic pain [20]) or the complexity of the conditions themselves (e.g., mental health conditions [21]), contemporary health systems should aim to utilize all resources at their disposal. An examination of the role of naturopaths in health care may present an opportunity to elucidate additional healthcare resources that are as yet unrecognized to mainstream health services and administrators. Thus, the primary intention of this research was to conduct an international survey of naturopathic practice and patient characteristics in order to gain insight to the breadth of practices and the type of clinical conditions routinely encountered in naturopathic visits.

## Methods

### Aim and study design

This cross-sectional survey aimed to describe the characteristics of typical naturopathic practices and their associated patients internationally.

### Setting

This study was conducted in naturopathic clinics in 14 countries within 4 world regions including the European (Portugal, United Kingdom, Switzerland, Spain), Americas (Canada, United States, Chile, Brazil), Western Pacific (Hong Kong, Australia, New Zealand) and African (South Africa).

### Participants

The study included naturopathic practitioners who were currently in practice and a member of a naturopathic association recognised by the WNF. Participants were required to have been in practice for at least five years, preferably seeing more than ten patients per week, and to have a computer terminal in their clinic. Naturopaths were excluded if they identified as practising within a specialised field (e.g. primarily focused on treating cancer or female reproductive conditions).

#### Recruitment

The World Naturopathic Federation shared an invitation from the research team with recognised naturopathic professional associations in preselected countries to forward on to their members. The countries included were identified by the World Naturopathic Federation as having sufficient infrastructure within the naturopathic profession to facilitate recruitment while also permitting global geographical distribution. Naturopathic practitioners interested in participating in the study accessed study documents online, including an information sheet and consent form. Following online screening, an automated email was sent to the research team who then emailed the participants a direct link to the online survey instrument. Participants were asked to prospectively complete the instrument for 20 consecutive cases. At the beginning of the survey the respondent was asked whether they had missed completing a survey about any previous patients and, if yes, queried to provide the reason the patient was not included.

#### Document translation

The invitations email, information sheet, screening instrument and survey were all initially drafted in English and then translated into French, Spanish, and Portuguese by native language speakers. The translated documents were then cross translated back to English by a second group of individuals. All translations were coordinated by the World Naturopathic Federation. The research team then checked the translations for accuracy with the original English documents. No discrepancies were found, and the translated documents were used in the study.

### Instrument

The survey was administered in four languages (English, French, Portuguese and Spanish) via Survey Gizmo. The survey included five domains: *(1) Patient sociodemographics, (2) Chief complaint/reason for care, (3) Interprofessional care, (4) Prescribed or recommended treatments and (5) Naturopathic interpretation of the health condition.*

#### Patient sociodemographics

Participants were asked to provide information about patients’ sex (male, female, non-binary) and age categories. No protected health information was collected in the survey.

#### Reason for visit

The survey queried the reason the patient visited with the naturopath on each occasion including: the visit type (initial visit or follow up consultation) and the nature of the primary complaint for which the patient was seeking assistance (i.e., chronic, acute, unsure). The naturopaths were also asked to identify the chief complaint for the patient, collected through a two-stage process. Firstly, participants were asked to select one of 17 system-based categories (e.g. gastrointestinal, respiratory, cardiovascular, autoimmune) for the patient’s presenting complaint. Upon selection of the system, survey logic populated a more specific list of conditions from which participants were required to select an option. For each list of conditions an ‘other’ option was also available which allowed respondents to manually enter a condition not included on the list. The list of conditions was developed based on an internationally available naturopathic clinical textbook [22].

#### Interprofessional care

Naturopaths were asked to identify any other health professionals (general practitioner, specialist doctor, allied health professional, complementary medicine practitioner, other health professionals) known to be providing care to the patient for the presenting complaint, where applicable.

#### Prescribed or recommended treatments

The survey also included items that asked naturopaths to identify the treatments prescribed or recommended to the patient based on a list of treatment categories (e.g. herbal medicines, dietary changes, acupuncture, lifestyle recommendations). The list was developed based on the most common therapies reported by the World Naturopathic Federation in the Naturopathic Roots Report [23].

#### Naturopathic interpretation of the health condition

Respondents were asked to indicate any other health systems they considered relevant or important to the management of the patient’s presenting complaint (e.g. endocrine system, gastrointestinal system, reproductive system). The same list of health systems used to capture data about the reason for the patient visit was employed for this survey item but respondents were able to select as many response options as they felt appropriate.

### Data management and analysis

Data were exported from Survey Gizmo as four separate Microsoft Excel spreadsheets. The data were merged into one dataset. All non-English responses to specific items were translated to English using a priori developed translations. All non-English open text responses were translated using Google Translate. All open text ‘other’ responses were cross-checked by a member of the research team (AS) against the available response options. For example, if a respondent selected ‘gastrointestinal system’ instead of ‘autoimmune condition’ for a patient presenting with coeliac disease then they would have entered this into the ‘other’ category. In these cases, the response was reallocated to the appropriate response. All data were then coded and imported into Stata 14.2 for analysis.

Descriptive statistics were tabulated as frequencies and percentages and chi square tests were used to test associations and compare groups. Treatment categories were collapsed into grouped variables for the following*: lifestyle and behavioural changes* (lifestyle, exercise, meditation, mind-body and rehabilitation exercise); *manual therapies* (massage, bodywork, acupuncture); *invasive treatments* (intravenous therapy, injection therapy, colonics, mesotherapy, chelation therapy, surgery); *other energetic medicines* (flower essences, tissue salts); *other traditional medicine systems* (traditional Chinese medicine, Ayurveda, humoral therapy, Unani medicine). Cumulative variables were also generated for the total number of treatment categories prescribed and the total number of other health systems considered by the naturopath to be relevant or important to the patient’s primary complaint.

### Ethical clearance

This project was approved by the Human Research Ethics Committee of the Endeavour College of Natural Health (#20181017).

## Results

A total of 56 naturopaths from 14 countries participated in the study, providing a mean of 15.1 cases each (SD 7.6) (see Table 1). The participants were drawn from countries representing the European, Americas, Western Pacific, and African world regions. The majority of naturopathic practitioners were female (62.5%) and their age was fairly evenly distributed although the most prevalent age group was 26–45 years (37.5%). Most participant naturopaths had been in practice for between 5 and 15 years (5–10 years, 44.6%; 11–15 years, 25.0%) and reported an average of 11–20 (35.7%) or 21–30 (21.4%) patient visits per week). In 4.1% of responses the participant naturopath indicated they had missed providing data for one of their patients.

**Table 1:** Participant characteristics (*n* = 56)

Characteristic	*n* (%)
Country	
*Australia*	6 (10.7)
*Brazil*	4 (7.1)
*Canada*	6 (10.7)
*Chile*	4 (7.1)
*Hong Kong*	3 (5.4)
*India*	7 (12.5)
*Nepal*	2 (3.6)
*New Zealand*	3 (5.4)
*Portugal*	4 (7.1)
*South Africa*	2 (3.6)
*Spain*	4 (7.1)
*Switzerland*	2 (3.6)
*United Kingdom*	3 (5.4)
*United States*	6 (10.7)
Gender	
*Female*	35 (62.5)
*Male*	21 (37.5)
Age	
*26–35 years*	11 (19.6)
*36–45 years*	21 (37.5)
*46–55 years*	11 (19.6)
*56–65 years*	11 (19.6)
*66 years or more*	2 (3.6)
Years in clinical practice	
*5–10 years*	25 (44.6)
*11–15 years*	14 (25.0)
*16–20 years*	5 (8.9)
*21–25 years*	6 (10.7)
*26 years*	6 (10.7)
Average number of patients per week	
*Less than 10*	9 (16.1)
*11–20*	20 (35.7)
*21–30*	12 (21.4)
*31–40*	8 (14.3)
*41–50*	4 (7.1)
*51 or more*	3 (5.4)
	Mean (SD; Range)
Average number of responses per participant	15.1 (7.6; 1–20)

Table 2 presents the sociodemographic characteristics of patient encounters (*n* = 851) as reported by the naturopathic practitioners. The majority of patients were reported by participant naturopaths as female (72.6%). All age categories were represented in the details reported by the participant naturopaths, with a similar proportion for 36–45 years (20.2%), 46–55 years (19.5%), and 56–65 years (19.3%). Approximately two thirds (67.0%) of patients were described as attending the participant naturopaths’ clinic for a follow up visit. A substantial majority (75%) of patients were considered by the participant naturopath to be presenting with a chronic health condition.

**Table 2:** Characteristics of patients as reported by participants (*n* = 851)

Sociodemographic characteristics	*n* (%)
Patient Sex (*n* = 851)	
*Female*	618 (72.6)
*Male*	233 (27.4)
Patient Age (*n* = 835)	
*Up to 5 years*	21 (2.5)
*6–12 years*	21 (2.5)
*13–17 years*	10 (1.2)
*18–25 years*	56 (6.7)
*26–35 years*	129 (15.5)
*36–45 years*	169 (20.2)
*46–55 years*	163 (19.5)
*56–65 years*	161 (19.3)
*66–75 years*	68 (8.1)
*76–85 years*	28 (3.4)
*86 years and over*	9 (1.1)
Visit type (*n* = 852)	
*First visit*	281 (33.0)
*Follow up visit*	571 (67.0)
Nature of the presenting complaint (*n* = 844)	
*Acute*	165 (19.6)
*Chronic*	633 (75.0)
*Unsure*	46 (5.5)

The primary reason for the patient visiting with the participant for naturopathic treatment was quite varied and is presented in Table 3. The most prevalent categories of health condition were musculoskeletal (18.5%), gastrointestinal (12.2%), and mental illness (11.0%). General wellness and prevention was also cited as a primary reason for patients consulting with the participant naturopath (6.7%). Eleven of the 17 categories of health conditions were described as the primary presenting complaint for between 6 and 3% of all patients. Patients reported by participant naturopaths as presenting with a musculoskeletal complaint as their primary concern, were most frequently identified as having chronic musculoskeletal pain (48.4%), injury (19.1%), or osteoarthritis (12.7%). Participant naturopaths indicated patients reporting a gastrointestinal condition were most frequently presenting with irritable bowel syndrome (31.7%), gastro-oesophageal reflux (17.3%), or food allergy, intolerance or sensitivity (16.4%). When asked to identify other physiological systems or health concerns being considered in the management of each patient’s health, the gastrointestinal system was most commonly selected (40.8%). Less common but still prevalent was general wellness and prevention (28.7%) and the endocrine system (23.8%). Participant naturopaths reported considering a mean of 2.4 other physiological systems or health categories for each individual case when developing a treatment plan (SD 1.7, range = 0–14) (data not shown in table).

**Table 3:** Primary health condition for which patients seek assistance and importance of other physiological systems in management of the patients case, as reported by naturopaths (*n* = 854)

Physiological system or category of the primary health condition	All responses n (%)	Specific primary health condition	Responses within the system or category n (%)	Considered important in the management of the primary condition [All responses, *n* (%)]
Musculoskeletal	158 (18.5)	Chronic musculoskeletal pain	76 (48.4)	151 (17.7)
		Injury	30 (19.1)	
		Osteoarthritis	20 (12.7)	
		Fibromyalgia or chronic fatigue syndrome	12 (7.6)	
		Sciatica	4 (2.6)	
		Other	15 (9.6)	
Gastrointestinal	104 (12.2)	Irritable bowel syndrome	33 (31.7)	348 (40.8)
		Gastro-oesophageal reflux	18 (17.3)	
		Food allergy/intolerance/sensitivity	17 (16.4)	
		Dysbiosis or parasites	8 (7.7)	
		Liver and biliary dysfunction and disease	6 (5.8)	
		Symptomatic constipation	3 (2.9)	
		Symptomatic diarrhoea	2 (1.9)	
		Inflammatory bowel disorders	1 (1.0)	
		Other	16 (5.8)	
Mental illness	93 (11.0)	Anxiety	26 (28.0)	133 (15.5)
		Depression	20 (21.5)	
		Stress or fatigue	17 (18.3)	
		Bipolar disorder	7 (7.5)	
		ADHD	6 (6.5)	
		Insomnia and other sleep disorders	5 (5.4)	
		ASD	2 (2.2)	
		Addiction	2 (2.2)	
		Other	8 (8.6)	
General wellness and prevention	57 (6.7)			245 (28.7)
Female reproductive	51 (6.0)	Menopausal symptoms	20 (39.2)	134 (15.7)
		Dysmenorrhoea and other menstrual complaints	12 (23.5)	
		Polycystic ovarian syndrome (PCOS)	9 (17.7)	
		Endometriosis	6 (11.7)	
		Fibroids and other benign tumours	3 (5.9)	
		Other	1 (2.0)	
Skin/Integumentary	44 (5.2)	Inflammatory skin conditions	25 (56.8)	79 (9.3)
		Acne vulgaris	11 (25.0)	
		Other	8 (18.2)	
Respiratory	43 (5.0)	Congestive respiratory disorders	23 (53.5)	71 (8.3)
		Respiratory tract infection	8 (18.6)	
		Asthma	6 (14.0)	
		Other	6 (14.0)	
Maternal health	43 (5.0)	Fertility	23 (54.8)	29 (3.4)
		Pregnancy	11 (26.2)	
		Preconception care	5 (11.9)	
		Lactation, breastfeeding and other postpartum care	3 (7.1)	
Neurological	43 (5.0)	Headache/migraine	24 (55.8)	67 (7.9)
		Neuralgia	7 (16.3)	
		Parkinson’s disease	3 (7.0	
		Paralysis and partial paralysis	3 (7.0)	
		Carpel tunnel syndrome	1 (2.3)	
		Other	5 (11.6)	
Endocrine	40 (4.7)	Thyroid abnormalities	22 (55.0)	203 (23.8)
		Type 2 diabetes	5 (12.5)	
		Adrenal insufficiency	5 (12.5)	
		Insulin resistance or metabolic syndrome	4 (10.0)	
		Other	4 (10.0)	
Cancer	39 (4.6)	Active, malignant cancer	17 (43.6)	29 (3.4)
		Post-cancer recovery, support and prevention	11 (28.2)	
		Management of cancer treatment side effects	5 (12.8)	
		Palliative care	3 (7.7)	
		Benign cancer	2 (5.1)	
		Other	1 (2.6)	
Cardiovascular	36 (4.2)	Hypertension	15 (41.7)	108 (12.7)
		Chronic venous insufficiency/poor circulation	9 (25.0)	
		Atherosclerosis and/or dyslipidemia	6 (16.7)	
		Stroke-related complaints	4 (11.1)	
		Other	2 (5.6)	
Weight management	34 (4.0)			147 (17.2)
Autoimmune	31 (3.6)	Systemic (e.g. SLE/lupus, Rheumatoid arthritis, ankylosing spondylitis)	18 (58.1)	74 (8.7)
		Gastrointestinal (coeliac, crohn’s, ulcerative colitis)	5 (16.1)	
		Nervous system (e.g. multiple sclerosis, myasthenia gravis)	3 (9.7)	
		Thyroid (e.g. Grave’s, Hashimoto’s)	2 (6.5)	
		Type 1 diabetes	2 (6.5)	
		Other	1 (3.2)	
Urogenital	21 (2.5)	Urinary tract infection	8 (38.1)	41 (4.8)
		Benign prostate hypertrophy	5 (23.8)	
		Kidney disease	3 (14.3)	
		Infections (candida, sexually transmitted infections)	3 (14.3)	
		Incontinence	2 (9.5)	
Ageing and cognition	10 (1.2)	Alzheimer’s disease or dementia	4 (40.0)	69 (8.1)
		Healthy ageing support	3 (30.0)	
		Other cognitive impairment	3 (30.0)	
Infectious disease	7 (0.8)	Lyme disease	3 (42.9)	27 (3.1)
		Epstein-barr virus	2 (28.6)	
		Other	2 (28.6)	

The most common treatment categories prescribed or recommended to patients by the participant naturopaths were dietary changes (60.5%), lifestyle and behaviour changes (56.9%), herbal medicines (54.2%) and nutritional supplements (52.1%) (see Table 4). Less common were acupuncture (27.2%), manual therapies (22.1%), homeopathy (22.0%), and counselling/psychotherapy (18.7%). Participant naturopaths reported prescribing or recommending a mean of 4.0 different treatment categories for each individual case (SD 1.8, range = 0–10) (data not shown in table).

**Table 4:** Categories of treatments prescribed to patients, as reported by naturopaths (*n* = 859)

Category of treatment prescribed	*N* (%)
Dietary changes	517 (60.5)
Lifestyle behaviour changes	486 (56.9)
Herbal medicines	463 (54.2)
Nutritional supplements	445 (52.1)
Acupuncture	233 (27.2)
Manual therapies	189 (22.1)
Homeopathy	188 (22.0)
Counselling and psychotherapy	160 (18.7)
Other energetic medicines	137 (16.0)
Testing or investigations	117 (13.7)
Hydrotherapy	115 (13.5)
Other Traditional medicine systems	110 (12.9)
Invasive therapies	58 (6.8)
Other treatments	222 (26.0)

Table 5 presents the details of other health professionals, as known to the participant naturopath, to be providing care to the same patient. Many patients were known to be receiving care from a general practitioner (43.2%) or a specialist medical practitioner (27.8%). Co-treatment by an allied health practitioner (12.4%) or a complementary medicine practitioner (10.9%) was less prevalent. According to the participant naturopaths, approximately one third of patients (33.0%) were known to be only consulting with the participant naturopath to manage their primary health concern.

**Table 5:** Other health professionals involved in treating the patient’s primary complaint, as reported by naturopaths (*n* = 859)

Health professional	*N* (%)
General practitioner	369 (43.2)
Specialist medical practitioner	237 (27.8)
Allied health practitioner	106 (12.4)
Complementary medicine practitioner	93 (10.9)
Other health professional	15 (1.8)
None	282 (33.0)

## Discussion

The results presented here represent the first known examination of international naturopathic practice. There are key findings with particular importance for the understanding of naturopathy in the context of contemporary healthcare practice and policy. Firstly, in all geographic settings naturopaths appear to treat patients with a diverse range of conditions and across all ages and gender. In many cases, they may also provide care to these patients without the involvement of other health professionals, indicating that they are practising as the primary care provider. These characteristics highlight the versatility of naturopathic practice as they align with the established definition of primary care in that it “addresses any health problem at any stage of a patient’s life cycle” [24]. Therefore, the scope of naturopathic practice may go beyond being solely classified as an additional complementary health care system alongside standard conventional care.

**Fig. 1:**
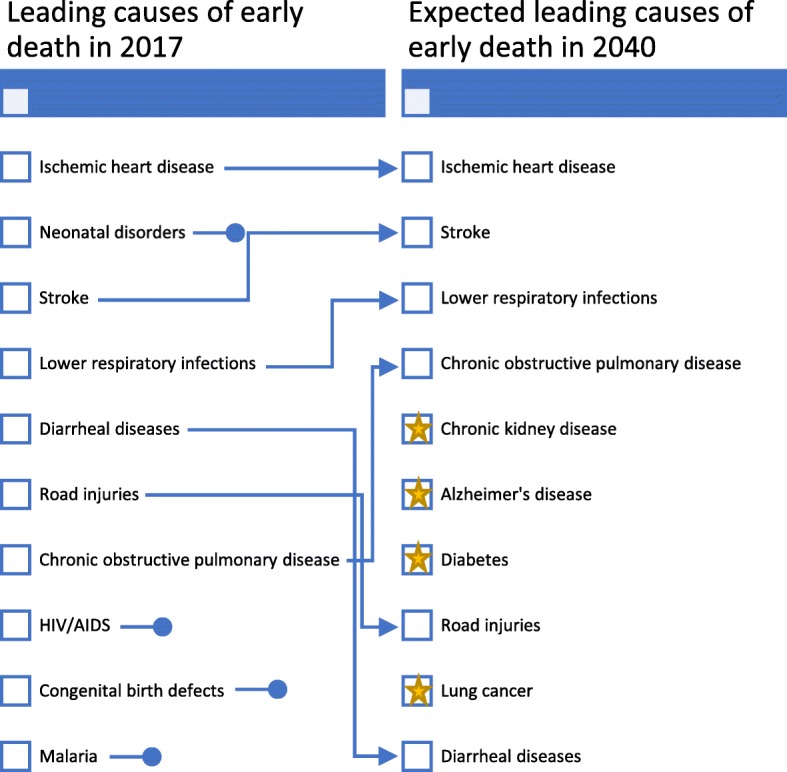
Leading causes of early death in 2017 and Expected leading causes of early death in 2040 (Source: Global Burden of Disease Study, 2017) [25]

**Fig. 2:**
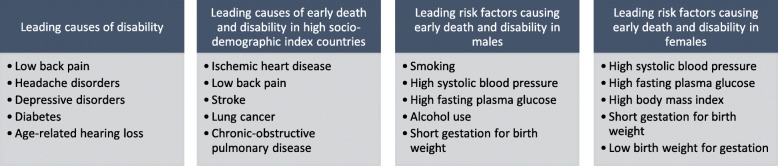
Early death and disability - causes and risk fctors in 2017 (Source: Global Burden of Disease Study, 2017) [25]

The patients visiting naturopaths in our study presented with conditions which not only demonstrate diversity, but also include conditions recognised as contributing strongly to the global burden of disease. The most recent Global Burden of Disease study reports ischaemic heart disease, stroke, respiratory infections and diarrheal diseases among the five leading causes of early death in 2017; all of which the participants in our study were treating (see Fig. 1) [25]. Four out of the five global leading causes of disability (low back pain, depressive disorders, headache and diabetes) were also among those reported by participants as the primary reason of their patient’s visit (see Fig. 2) [26]. Furthermore, nine of the ten leading causes of early death expected in 2030 are also featured in the list of conditions for which patients were described as seeking treatment for from a naturopathic practitioner [27]. The current and potential future contributions being made by naturopathic practitioners toward mitigating the effects of these conditions on the global burden of disease are, at present, unclear and deserving of further attention. Many of these conditions are noncommunicable diseases (NCDs) with high quality established evidence for preventive care and health promotion counselling to reduce established risk factors [28]. Considering the fact naturopaths are treating NCDs, measuring and quantifying their contributions to reduced disease burden and impact on national medical expenses for countries warrants further investigation.

A prominent feature for the majority of the NCDs commonly treated by naturopaths in this study is the importance of diet and lifestyle as evidence-based primary prevention, particularly for cardiovascular disease [26, 28], diabetes [29], lung cancer [30], chronic kidney disease [31], and chronic obstructive pulmonary disease [32], with additional emerging evidence for Alzheimer’s disease [33] and lower respiratory tract infections [34]. Interestingly for the latter, prevention of lower respiratory tract infections has been linked to various factors including improved sleep, dietary modifications, improved immune function, and psychological support suggesting that a holistic approach to clinical care is required [34]. Holism is integral to naturopathic philosophy, and preventive care is reflected in the core naturopathic principle of *Disease prevention and health promotion* [3]. While primary prevention is a global priority for the health conditions causing early death and disability, it is also worth noting that primary care practitioners may be challenged to accommodate preventive health care service delivery within their usual care load [35]. As such, naturopathic practitioners may be an untapped health resource in many health systems which can relieve the burden on primary care physicians [36]. While our study does not detail the specific prevention, screening or treatment methods used by the clinician, the data suggests they were considering body weight, metabolic disorders, and diet and lifestyle changes in the context of patient care; all of which are important modifiable risk factors for morbidity and mortality [25]. Further clinical research that explores the patient outcomes of naturopathic care for the prevention of these globally important conditions is urgently needed.

This study also describes unique and diverse treatments employed by naturopathic practitioners as part of routine patient care, that are not delivered, or counselled on, by other types of clinicians. While some treatments were prescribed or recommended in most cases (dietary modifications, lifestyle changes, herbal medicines, nutritional products), there were many other treatment categories reported. In addition, the study results evidence that the clinicians were employing multiple treatments in the care of an individual patient. This finding aligns with a report by the WNF describing the content of naturopathic curriculum worldwide which noted that clinical nutrition (dietary prescription), applied nutrition (individualised nutritional product prescription), and botanical medicine (herbal medicine) are taught in more than 90% of recognised naturopathic programs internationally [23]. According to the WNF Roots Report [23], lifestyle counselling is not commonly taught within naturopathic curricula, but was still listed in more than 70% of cases in our study. This discrepancy between the use of lifestyle prescription in practice and the frequency of its inclusion in naturopathic curricula highlights a need for further investigation of the content and impact of tacit content and the need for naturopathic educational organisations to address any gaps in training in some countries. Given the importance of lifestyle interventions in prevention and management of NCDs and the findings of our study this is an important area of naturopathic care.

The variance in therapeutic tools employed by naturopathic practitioners in our study also reinforces the position held by the WNF that the naturopathic profession is a traditional system of medicine defined by its philosophies and principles [3] rather than by specific practices. For example, the frequency with which naturopathic practitioners in our study identified considering other physiological systems and health concerns when managing an individual’s primary presenting complaints may demonstrate the clinician’s application of core naturopathic principles such as: *Treat the whole person*, *Treat the cause*; and *Disease prevention and health promotion* [3]. Yet the treatments employed by naturopathic practitioners may vary across countries when applying these principles. This difference in application may be due to the influence of various local social, cultural, and legislative frameworks [15]; a factor contributing to the complexity of global naturopathic practice. These local differences may also impact on care provision and whether naturopathy is being accessed in a complementary or primary care context. Previous research has indicated the potential for naturopaths to be functioning as primary care practitioners [37]. Legislative frameworks in specific states in the United States and Canada already clearly position naturopathic practitioners as primary care physicians [15]. The extent to which a primary care capacity is filled by naturopathic practitioners may vary in different countries but is a topic worthy of further exploration.

Equally, research evaluating the effectiveness of naturopathic care ideally should employ a whole practice research designs that accounts for the complex treatment mix and individualised treatment approach characteristic of naturopathic practice. Available evaluations of outcomes from naturopathic practices suggest naturopathic care may improve the outcomes of patients with cardiovascular conditions, diabetes, chronic pain, autoimmune disease, mental illness, and chronic obstructive pulmonary disease [38]. While there may be evidence supporting the application of specific treatments used by naturopathic practitioners in the management of some of these conditions [39–48], further research is required to fully quantify the impact and effectiveness of naturopathic care on the clinical outcomes across the diverse health complaints clinicians appear to be treating. Additionally, while all of conditions identified in this recent review are among those reported in our study as the primary reason patients have visited with naturopathic practitioners, there are also numerous conditions for which naturopathic care has not been examined.

### Limitations

As usual, the findings reported here should be viewed within the context of the study’s limitations. While this is the most comprehensive global study to date examining the characteristics of naturopathic clinical practice, it cannot be viewed as generalizable to the entirety of the international naturopathic profession. Instead, this study provides preliminary data that should be examined in more detail or in larger, more focused studies. The diversity of naturopathic practice in different geographical areas will be affected by social, cultural and legislative influences which should be carefully considered within unique national and regional settings. However, the scope and ethical constraints of this study did not permit inter-regional analysis. The recruitment frame, limited to members of professional associations, also biases the results toward those naturopaths who may be more academic, transparent and/or generally professional in their practices. Equally, the requirement for participants to have a computer at their clinical location may have also introduced a bias, as naturopaths without access to a computer in their clinic may have other practice differences compared to those that do. Due to the heterogeneity of practice locations and the pilot nature of this study, it was decided to have a few practitioners from each country only. However, as described by the Agency for Health Research and Quality, a level of representativeness can be afforded by a practice-based research study conducted in a minimum of five locations and with at least 15 participating clinicians [49]. The survey data also relies on self-report, which may result in additional bias. Equally, some survey items required participants to report on patient characteristics and the accuracy of this data was not independently confirmed by the researchers. Similarly, patients were not contacted directly for confirmation of their chief complaints nor their engagement with other health care practitioners involved in their care. Despite these limitations, this study offers an important contribution to the understanding of naturopathic practice at a global level.

## Conclusions

Naturopathic practitioners provide health care for diverse health conditions across the life course. Patients are consulting with naturopathic practitioners for support with health conditions of global importance and there is emerging evidence to suggest naturopathic care may benefit individuals with some of these conditions. Overall, this study suggests naturopathic practitioners may represent an aspect of primary care and disease prevention that is accessed by patients around the world. The global population would benefit from researchers and policy makers paying closer attention to the potential risks, benefits, challenges and opportunities of the provision of naturopathic care within the community.

## Data Availability

The datasets generated and analysed during the current study are not publicly available due to intellectual property agreements but are available from the corresponding author on reasonable request.

## Abbreviations

D.C: District of Columbia
NCDs: Non-communicable diseases
T&CM: Traditional and complementary medicine
WHO: World Health Organisation
WNF: World Naturopathic Federation
